# Combined organ xenotransplantation: from graft function to cross-species homeostasis

**DOI:** 10.1093/lifemedi/lnag034

**Published:** 2026-09-21

**Authors:** Kai Xing, Yuqi An, Yicheng Ling, Xiao Chen, Jiangping Song

**Affiliations:** Sanya Institute of China Agricultural University, Sanya 572025, China; Department of Cardiac Surgery, Fuwai Hospital, National Center for Cardiovascular Diseases, Chinese Academy of Medical Sciences and Peking Union Medical College, Beijing 100037, China; State Key Laboratory of Cardiovascular Disease, Fuwai Hospital, National Center for Cardiovascular Diseases, Chinese Academy of Medical Sciences and Peking Union Medical College, Beijing 102300, China; Beijing Key Laboratory of Xenotransplantation, Fuwai Hospital, National Center for Cardiovascular Diseases, Chinese Academy of Medical Sciences and Peking Union Medical College, Beijing 102300, China; State Key Laboratory of Cardiovascular Disease, Fuwai Hospital, National Center for Cardiovascular Diseases, Chinese Academy of Medical Sciences and Peking Union Medical College, Beijing 102300, China; Beijing Key Laboratory of Xenotransplantation, Fuwai Hospital, National Center for Cardiovascular Diseases, Chinese Academy of Medical Sciences and Peking Union Medical College, Beijing 102300, China; First Teaching Hospital of Tianjin University of Traditional Chinese Medicine, Tianjin 300110, China; Sanya Institute of China Agricultural University, Sanya 572025, China; Department of Cardiac Surgery, Fuwai Hospital, National Center for Cardiovascular Diseases, Chinese Academy of Medical Sciences and Peking Union Medical College, Beijing 100037, China; State Key Laboratory of Cardiovascular Disease, Fuwai Hospital, National Center for Cardiovascular Diseases, Chinese Academy of Medical Sciences and Peking Union Medical College, Beijing 102300, China; Beijing Key Laboratory of Xenotransplantation, Fuwai Hospital, National Center for Cardiovascular Diseases, Chinese Academy of Medical Sciences and Peking Union Medical College, Beijing 102300, China; Sanya Institute of China Agricultural University, Sanya 572025, China; State Key Laboratory of Cardiovascular Disease, Fuwai Hospital, National Center for Cardiovascular Diseases, Chinese Academy of Medical Sciences and Peking Union Medical College, Beijing 102300, China; Beijing Key Laboratory of Xenotransplantation, Fuwai Hospital, National Center for Cardiovascular Diseases, Chinese Academy of Medical Sciences and Peking Union Medical College, Beijing 102300, China; Sanya Institute of China Agricultural University, Sanya 572025, China; Department of Cardiac Surgery, Fuwai Hospital, National Center for Cardiovascular Diseases, Chinese Academy of Medical Sciences and Peking Union Medical College, Beijing 100037, China; State Key Laboratory of Cardiovascular Disease, Fuwai Hospital, National Center for Cardiovascular Diseases, Chinese Academy of Medical Sciences and Peking Union Medical College, Beijing 102300, China; Beijing Key Laboratory of Xenotransplantation, Fuwai Hospital, National Center for Cardiovascular Diseases, Chinese Academy of Medical Sciences and Peking Union Medical College, Beijing 102300, China; Clinical Research Institute, Chinese Academy of Medical Sciences, Beijing 100730, China; Faculty of Clinical Medicine, Peking Union Medical College, Beijing 100730, China; Shenzhen Key Laboratory of Cardiovascular Disease, Fuwai Hospital, Chinese Academy of Medical Sciences, Shenzhen 518057, China

Combined organ xenotransplantation represents a new frontier beyond single-organ replacement, testing whether multiple porcine organs can be integrated into human physiological homeostasis. Here, a six-gene-edited pig liver and bilateral kidneys supported short-term function in a human decedent model, while revealing the remaining challenges of immune regulation, coagulation control, and long-term cross-species adaptation.

Organ shortage has long limited treatment options for patients with end-stage organ failure, and recent advances have brought xenotransplantation closer to clinical translation. Genetically engineered porcine kidneys, hearts, and auxiliary livers have shown transient function in human recipients or decedent models, supporting porcine organs as potential alternative grafts [1–5]. However, previous studies have largely focused on single-organ or auxiliary transplantation, leaving a fundamental question unresolved: whether multiple xenogeneic organs can jointly maintain human physiological homeostasis rather than merely support individual organ function.

Combined organ xenotransplantation therefore represents a higher-order challenge. The liver and kidney are highly interconnected regulators of systemic metabolism, coagulation, and internal homeostasis. Replacing both organs simultaneously tests whether porcine grafts can establish a functional cross-species network within the human body. The recent report of orthotopic whole-liver and bilateral-kidney xenotransplantation advances the field from evaluating isolated graft function toward assessing integrated physiological adaptation.

In this study, a whole liver and bilateral kidneys from a six-gene-edited Bama miniature pig were orthotopically transplanted into a human decedent recipient and monitored for about 5 days. The donor pig carried knockouts of *GGTA1*, *CMAH*, and *B4GALNT2* to remove major xenogeneic carbohydrate antigens, together with *hCD46*, *hCD55*, and *hTBM* transgenes to regulate complement and coagulation pathways. A limitation of the study is that quantitative characterization of human transgene expression in donor tissues, particularly in liver and kidney vascular endothelial cells, was not provided. After transplantation, the porcine liver produced detectable bile, portal venous flow remained stable, and liver function tests showed no evidence of extensive hepatocellular injury. Following bilateral kidney transplantation, serum creatinine and urea levels decreased, with gradual improvement in estimated glomerular filtration rate. Immunopathological analysis showed no substantial complement or immunoglobulin deposition typical of classical hyperacute rejection [1]. Together with single-cell RNA sequencing (scRNA-seq), proteomic, and metabolomic analyses, these findings provide evidence that combined porcine liver-kidney grafts can achieve short-term functional activity in a human decedent model, supporting technical feasibility without establishing long-term tolerance or durable graft survival.

The clinical value of this study lies not simply in increasing the number of grafts, but in testing whether multiple porcine organs can establish a transient functional network across species. In single-organ xenotransplantation, functional recovery can often be assessed through organ-specific readouts, such as serum creatinine, urine output, bile production, or biochemical markers. In contrast, combined liver-kidney xenotransplantation evaluates whether these functions can collectively maintain systemic homeostasis and prevent interactions among complement activation, coagulation disturbance, inflammation, and metabolic imbalance. This systems-level perspective distinguishes combined organ xenotransplantation from previous single-organ or auxiliary models. However, the translational priorities of the field should also be considered. Except for kidney xenotransplantation, long-term survival of individual porcine organs remains limited in primate and human studies. Therefore, human decedent multi-organ models should be viewed primarily as platforms for mechanistic investigation and feasibility assessment, rather than replacements for efforts aimed at overcoming the barriers limiting durable single-organ xenograft survival.

The immunological findings also extend current views of early xenograft injury. In this study, the donor gene edits and perioperative immunosuppression appeared to prevent classical hyperacute rejection in the short term. However, single-cell RNA sequencing (scRNA-seq) showed rapid infiltration of human immune cells into both liver and kidney grafts [1, 3]. *S100A12^+^* inflammatory myeloid or neutrophil-like cells expanded markedly and occupied central positions in inferred cell-cell communication networks. Enrichment of an *ADGRE*-related signaling axis suggests that early xenogeneic responses may involve myeloid activation, endothelial crosstalk, inflammatory amplification, and thromboinflammation.

This observation broadens the understanding of early xenograft injury beyond classical hyperacute rejection. In combined liver-kidney xenotransplantation, extensive microvascular networks make endothelial activation, granulocyte adhesion, platelet-neutrophil interactions, and local coagulation potential contributors to impaired organ perfusion and systemic hemostatic imbalance. Future strategies may therefore need to extend beyond B-cell, T-cell, and complement blockade to target myeloid inflammation, neutrophil extracellular trap (NET) formation, and endothelial protection. Interpretation of *S100A12* expression requires caution. *S100A12* is not neutrophil-specific and may also be expressed by inflammatory monocytes or other myeloid subsets. Although *S100A12* immunohistochemistry supports the presence of *S100A12*^+^ cells within grafts, it cannot define their lineage or spatial context. In addition, the identification of neutrophil-like cells from the peripheral blood mononuclear cell (PBMC) fraction obtained by density-gradient centrifugation requires careful interpretation. Although low-density neutrophils can co-isolate with PBMC under inflammatory conditions, the observed signal may also be influenced by cell aggregation, platelet contamination, or doublets. Moreover, the six-gene-edited donor pig used in this study lacked human CD47, and impaired CD47-SIRPα compatibility may have contributed to enhanced innate immune activation involving macrophages, neutrophils, and natural killer cells. Therefore, *S100A12*^+^ inflammatory myeloid responses should be considered candidate mediators of early xenograft injury rather than confirmed causal drivers.

Metabolomic profiling provides a complementary systems-level perspective on combined liver-kidney xenotransplantation. The authors showed that post-transplant bile, urine, and plasma metabolomic profiles remained correlated with recipient baselines and demonstrated temporal continuity, suggesting that combined porcine liver-kidney grafts did not cause overt systemic metabolic collapse during the observation period. Changes in pathways related to bile acids, bilirubin metabolism, purine and pyrimidine metabolism, the urea cycle, lipid metabolism, and amino acid derivatives were consistent with graft activity and early cross-species metabolic adaptation. However, the interpretation that metabolism “shifted toward a human state” requires caution. Plasma metabolites are strongly influenced by the recipient circulation, fluid management, medications, and perioperative conditions. Therefore, similarity to human baseline does not necessarily indicate complete humanized metabolic reprogramming by porcine organs. Bile and urine may provide more direct evidence of liver and kidney graft function, but remain affected by ischemia-reperfusion injury, organ handling, and postoperative management. These findings therefore support short-term metabolic continuity and recipient-environment-driven adaptation rather than a fully established human metabolic state.

Similarly, the increase in circulating human albumin requires careful interpretation. Because the donor pig did not carry a human albumin transgene, increased human albumin signals are unlikely to represent *de novo* synthesis by the porcine liver. Instead, they may reflect residual recipient albumin, perioperative albumin or plasma administration, redistribution, or limitations in species-specific peptide discrimination. Future studies incorporating species-resolved proteomics, detailed transfusion records, and appropriate allotransplant controls will be needed to distinguish true cross-species metabolic adaptation from general postoperative responses.

The immunosuppressive regimen also warrants discussion. The study used rituximab, rabbit anti-thymocyte globulin, high-dose glucocorticoids, eculizumab, anti-CD154 monoclonal antibody (mAb), tacrolimus, and mycophenolic acid, targeting major early risks in xenotransplantation, including natural antibody responses, complement effector formation, lymphocyte priming, and inflammation [1, 2, 4, 5]. The authors also reported lower tacrolimus exposure than in previous xenotransplantation studies. If confirmed in larger studies, this finding may suggest an immunomodulatory effect of liver-inclusive transplantation, possibly through antigen adsorption, immune remodeling, or regulatory responses. For now, however, the short observation period does not allow lower tacrolimus exposure to be interpreted as evidence of immune tolerance. More importantly, this regimen should be viewed as a feasibility strategy rather than a mature long-term protocol. C5 blockade does not prevent upstream C3 cleavage, C4d deposition, or complement-mediated endothelial activation. Rituximab also cannot eliminate long-lived plasma cells or pre-existing anti-porcine antibodies. Although anti-CD154 blockade is central to prolonged xenograft survival, different anti-CD154 reagents are not equivalent. Conventional Fc-intact anti-CD154 antibodies can induce platelet activation and increase thromboembolic risk, whereas engineered or Fc-silent reagents may have different safety profiles. Therefore, the Fc properties, platelet-activating potential, pharmacokinetics, and timing of the anti-CD154 antibody require further clarification. For liver xenotransplantation, recipient platelet consumption and consumptive coagulopathy remain key barriers to long-term survival. In combined liver-kidney transplantation, platelet sequestration, microvascular coagulation, and coagulation factor consumption may impair not only hepatic perfusion but also the renal microcirculation. Platelet dynamics and coagulation safety should therefore be monitored as core endpoints. More broadly, this regimen also does not directly address the myeloid, neutrophil, NET, platelet, or endothelial pathways highlighted by the single-cell data. Preventing hyperacute rejection is only the first step; durable multi-organ xenotransplantation will require sustained control of delayed humoral responses, complement-coagulation-myeloid interactions, infection risk, and metabolic stability.

The human decedent model is valuable for translational research because it allows porcine organs to be tested in a human complement, coagulation, immune, and metabolic environment while avoiding the immediate ethical risks of living-recipient trials. However, brain death itself can trigger systemic inflammation, endocrine disruption, hemodynamic instability, and immune changes [3–5]. In addition, a single case with an about 5-day observation period cannot adequately address acute humoral rejection, cellular rejection, chronic rejection, opportunistic infection, progressive consumptive coagulopathy, or long-term metabolic adaptation. This study should therefore be viewed as an in-human feasibility model rather than a clinical endpoint.

Overall, this study advances xenotransplantation from single-graft survival toward systemic physiological integration. Multi-gene editing, intensive immunosuppression, and close monitoring enabled a porcine whole liver and bilateral kidneys to maintain short-term function in a decedent model without classical hyperacute rejection. The next question is whether these organs can integrate stably into human physiology over longer periods (Fig. 1). This will require improved genetic engineering, precise immune and coagulation control, longer observation, reproducible cohorts, and a clearer clinical path as bridge therapy for end-stage multi-organ failure or durable replacement in selected patients. Ultimately, the study provides a framework for testing whether multiple porcine organs can contribute to integrated human homeostasis.

**Figure 1.**
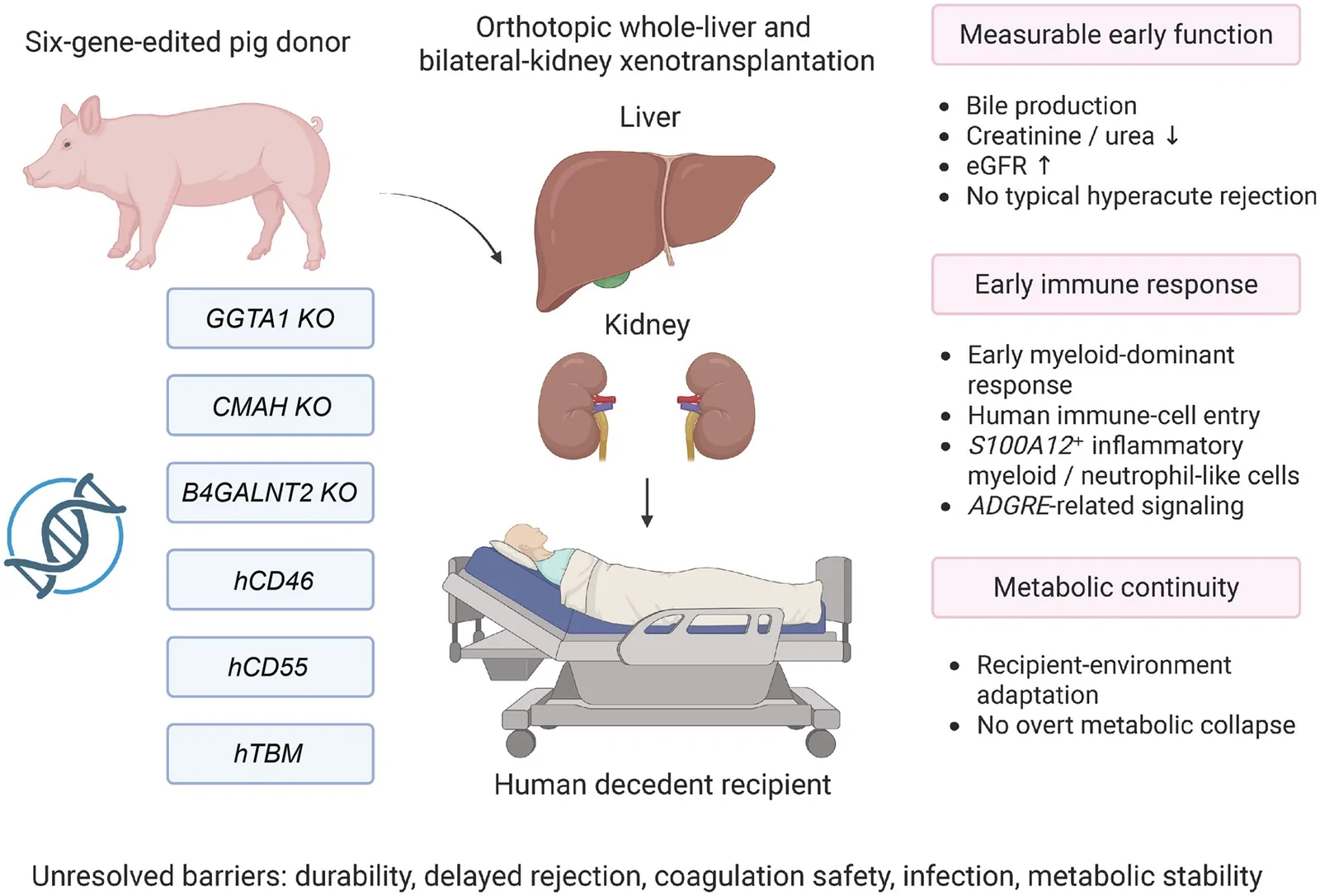
Conceptual framework of combined organ xenotransplantation. A six-gene-edited pig donor provides an orthotopic whole-liver and bilateral-kidney xenotransplant for a human decedent recipient. The model enables assessment of early graft function, immune activation, and metabolic continuity, while highlighting unresolved barriers including durability, delayed rejection, coagulation safety, infection, and metabolic stability. This figure was created with BioRender.com under an authorized license.
